# Fetal extracellular matrix nerve wraps locally improve peripheral nerve remodeling after complete transection and direct repair in rat

**DOI:** 10.1038/s41598-018-22628-8

**Published:** 2018-03-14

**Authors:** Tanchen Ren, Anne Faust, Yolandi van der Merwe, Bo Xiao, Scott Johnson, Apoorva Kandakatla, Vijay S. Gorantla, Stephen F. Badylak, Kia M. Washington, Michael B. Steketee

**Affiliations:** 1 Department of Ophthalmology, School of Medicine, University of Pittsburgh, Pittsburgh, PA USA; 2 McGowan Institute for Regenerative Medicine, University of Pittsburgh, Pittsburgh, PA USA; 3 Center for Neuroscience, University of Pittsburgh, Pittsburgh, PA USA; 4 Swanson School of Engineering, Department of Bioengineering, Pittsburgh, PA USA; 5 Department of Surgery, School of Medicine, University of Pittsburgh, Pittsburgh, PA USA; 6 Department of Plastic Surgery, School of Medicine, University of Pittsburgh, Pittsburgh, PA USA; 7 VA Pittsburgh Healthcare System Pittsburgh, Pittsburgh, PA USA; 8 Plastic Surgery, Xijing Hospital, The Fourth Military Medical University, Xi’an, China

## Abstract

In peripheral nerve (PN) injuries requiring surgical repair, as in PN transection, cellular and ECM remodeling at PN epineurial repair sites is hypothesized to reduce PN functional outcomes by slowing, misdirecting, or preventing axons from regrowing appropriately across the repair site. Herein this study reports on deriving and analyzing fetal porcine urinary bladder extracellular matrix (fUB-ECM) by vacuum assisted decellularization, fabricating fUBM-ECM nerve wraps, and testing fUB-ECM nerve wrap biocompatibility and bioactivity in a trigeminal, infraorbital nerve (ION) branch transection and direct end-to-end repair model in rat. FUB-ECM nerve wraps significantly improved epi- and endoneurial organization and increased both neovascularization and growth associated protein-43 (GAP-43) expression at PN repair sites, 28-days post surgery. However, the number of neurofilament positive axons, remyelination, and whisker-evoked response properties of ION axons were unaltered, indicating improved tissue remodeling per se does not predict axon regrowth, remyelination, and the return of mechanoreceptor cortical signaling. This study shows fUB-ECM nerve wraps are biocompatible, bioactive, and good experimental and potentially clinical devices for treating epineurial repairs. Moreover, this study highlights the value provided by precise, analytic models, like the ION repair model, in understanding how PN tissue remodeling relates to axonal regrowth, remyelination, and axonal response properties.

## Introduction

Peripheral nerve (PN) injury can lead to permanently lost sensation, motor control, and neuropathic pain. Though PN axons can regrow to restore function, the severity, location, and repair method influences local tissue remodeling and axon regrowth across the repaired injury site. PN injuries requiring surgical epineurial repair are the most challenging, as in anastomosis or coaptation to reconnect severed nerve trunks, and functional outcomes are generally poor despite advances in microsurgical nerve reconstruction techniques^1,2^. Epineurial repairs can locally increase the pro-inflammatory innate immune response and tissue remodeling that forms scar tissue. Increased scarring is hypothesized to slow and misdirect axon regrowth and increase deficits in tactile and sensimotor control^3^, neuroma formation and persistent pain^4^, and neuronal death^5^. However, whether improving tissue remodeling at epineurial repair sites is sufficient to increase functional axon regrowth is unclear.

Nerve wraps have been used to treat epineurial repairs since the early 1900s^6^ and several nerve wraps made from purified collagen^7^ or amniotic membrane^8^ are FDA approved, commercially available, and used clinically to treat both direct and indirect PN repairs, including neurolysis, direct end-to-end anastomosis, and coaptation between nerve trunks and autologous nerve grafts. Generally, commercial nerve wraps are semipermeable, resorbable collagen-based sheets. By wrapping PN epineurial repairs, nerve wraps protect the repair site during the initial healing phase. Moreover, these wraps are hypothesized to improve outcomes by providing a positive microenvironment that can reduce scarring^9^, scar-based ischemic adhesions^10^, neuroma formation^11^, and, in some cases, increase functional axonal reinnervation^8,12^. However, after complete PN transection and repair, the number of axons correctly reinnervating their appropriate target tissues often remains low, particularly in facial nerves, like the trigeminal nerve^13^. Moreover, whether nerve wrap associated functional improvements are due to improved tissue remodeling at epineurial repair sites that in turn increase appropriate functional axon regrowth across the repairs or due to reduced comorbidities remains unclear.

To determine how tissue remodeling influences functional axonal regrowth, we developed a nerve wrap from fetal porcine urinary bladder extracellular matrix (fUB-ECM). We hypothesize extracellular matrix (ECM) based nerve wraps derived from pro-regenerative xenogeneic tissues, like fetal porcine urinary bladder, will improve on currently available nerve wraps, mechanically, biochemically, and in tunablility. ECM bioscaffolds harbor numerous identified bioactive factors that can stimulate constructive tissue remodeling to reduce scar tissue formation, and to restore tissue appropriate function^14^ in all four major tissue types, including connective^15^, skeletal muscle^16^, epithelial^17^, and even nervous tissues^18,19^, with ECM derived from younger tissues often more efficacious^20,21^. Mechanistically, ECM bioscaffolds and/or ECM derived factors have been shown to positively modulate the innate immune response^22,23^, and increase site-appropriate tissue remodeling over scarring^24^, increase neovascularization^25,26^, and promote Schwann cell migration and differentiation^27^, neurogenesis^28^, and neurodifferentiation^29^. Moreover, ECM is a highly tunable platform that can be modified mechanically and biochemically based on the nature and the scope of the injury^30–32^. However, using naturally derived, acellular ECM-based nerve wraps for epineurial PN repairs is virtually unreported.

This study’s objectives were to decellularize fetal porcine urinary bladder using vacuum assisted decellularization (VAD)^33^ to produce acellular fUB-ECM bioscaffolds, to construct single layer fUB-ECM nerve wraps and characterize their initial biochemical and material properties, and to determine fUB-ECM nerve wrap biocompatibility and bioactivity *in vivo*. Biocompatibility and bioactivity were tested in an infraorbital nerve (ION) transection and direct end-to end repair (cut-repair) model. The ION is a flat, purely sensory nerve that innervates the rodent whisker pad and relays whisker-evoked mechanoreceptor stimuli from the whisker sinus to the barrel cortex with a gross topographical organization. Within the whisker sinus, ION axons innervate two mechanoreceptor classes, slowly adapting (SA) Merkel and rapidly adapting (RA) lanceolate receptors. This model permits precise electrophysiological analysis of ION axon reinnervation patterns and whisker-evoked axonal response properties in the trigeminal ganglion. Moreover, unlike other peripheral nerves, the repaired ION in rodents fails to recover completely^34,^ similar to the trigeminal nerve in humans^13^, leading to permanently lost neurologic function^35,36^. Incomplete recovery enables studies on PN tissue remodeling and axon regrowth, guidance, remyelination, and functional reinnervation. *In vivo*, fUB-ECM nerve wraps were biocompatible and durable over 28-days and bioactive, increasing epi- and endoneurial remodeling, neovascularization, and growth associated protein-43 (GAP-43) expression at ION repair sites. However, axon regrowth across repair sites, remyelination, and whisker-evoked response properties were unchanged, indicating positive tissue remodeling at PN repair sites is insufficient to predict axonal regrowth, remyelination, and functional reinnervation.

## Results

### VAD decellularized fetal urinary bladder ECM

**Figure 1:**
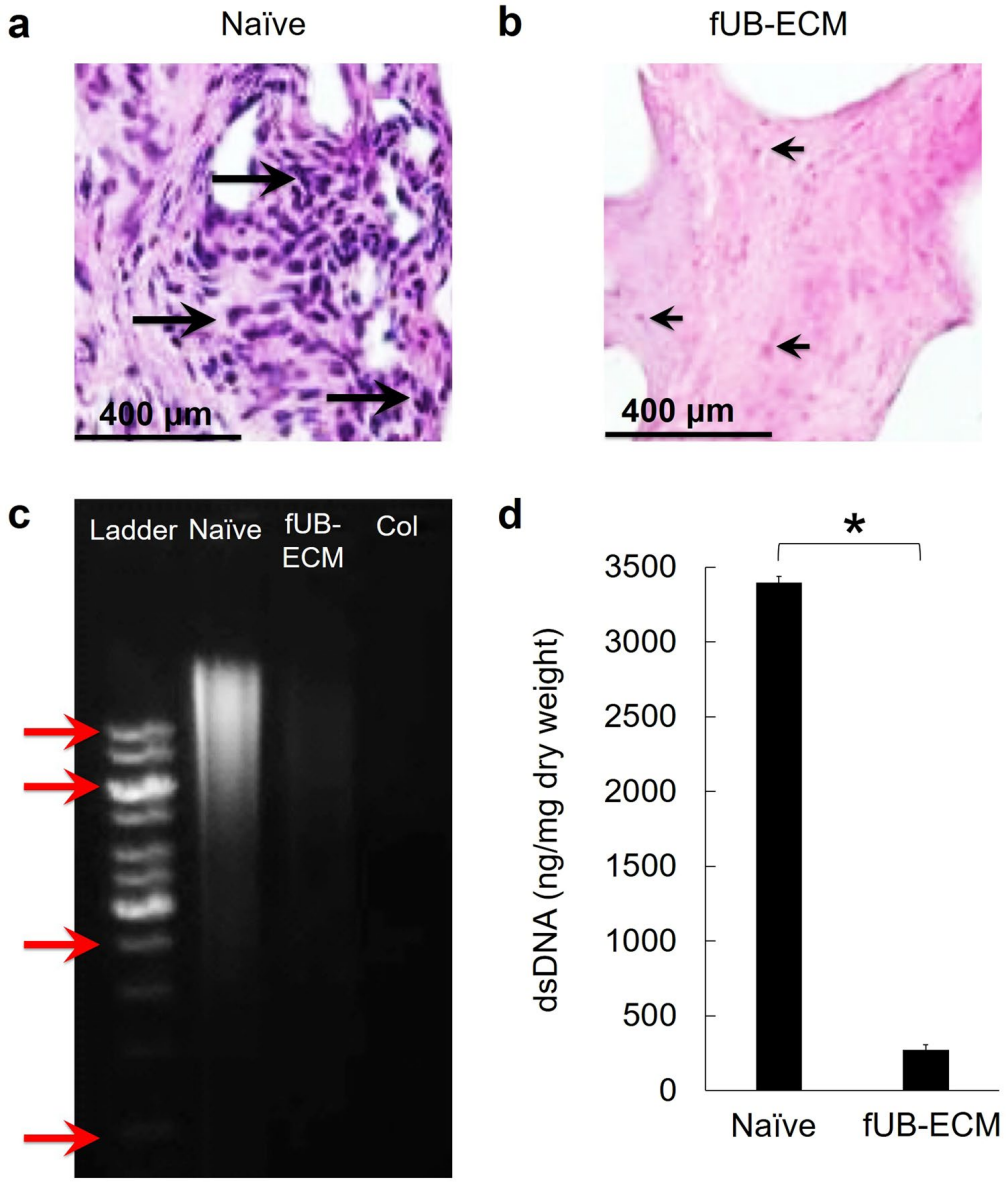
Fetal porcine urinary bladder extracellular matrix (fUB-ECM) was derived by vacuum assisted decellularization (VAD). (**a**) H&E staining showed hematoxylin positive DNA containing nuclei (dark purple, long arrows) in naïve fetal urinary bladder. (**b**) After VAD, hematoxylin staining was largely absent, revealing eosin positive nuclear associated proteins (pink, short arrows) in the resulting fUB-ECM. (**c**) Gel electrophoresis showed obvious high molecular weight DNA in naïve fetal urinary bladder (Naïve) was highly reduced in fUB-ECM. Col indicates the purified collagen control and the ladder arrows indicate (top to bottom) 1500 bp, 1000 bp, 500 bp, and 200 bp. (**d**) Compared to naïve, PicoGreen quantification showed double stranded DNA (dsDNA) was approximately 12-fold or 92% lower in fUB-ECM. Data represent n = 4 naïve fetal urinary bladders and n = 4 separate fUB-ECM VAD preparations. *p < 0.0001.

Fetal urinary bladders (fUB) were decellularized by VAD^29^ (Fig. 1). Naïve fUB retained hematoxylin positive DNA and nuclei (Fig. 1a). After VAD, hematoxylin positive nuclei were absent from fUB-ECM but eosin positive acidophilic nuclear-associated protein staining remained (Fig. 1b). Compared to naïve fUB and purified collagen, residual DNA was largely undetected by gel electrophoresis in fUB-ECM (Fig. 1c and Fig. S1). Quantification of double stranded DNA (dsDNA) showed dsDNA was reduced by 92% from 3397 ± 42 ng/mg dry weight in naïve fUB to 273 ± 35 ng/mg dry weight in fUB-ECM (Fig. 1d).

### Collagen, sulfated glycosaminoglycan, and hyaluronic acid

**Figure 2:**
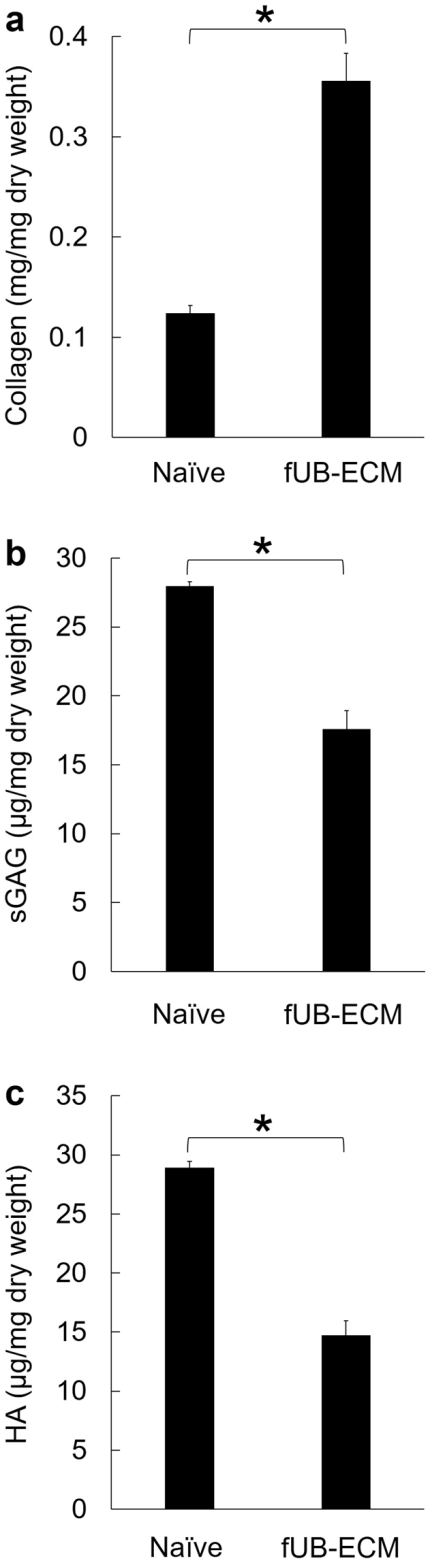
Compared to naïve fetal urinary bladder (Naïve), VAD increased (**a**) collagen content and decreased (**b**) sulfated glycosaminoglycan (sGAG) and (**c**) hyaluronic acid (HA) content. Data represent n = 3 naïve fetal urinary bladders and n = 3 separate fUB-ECM VAD preparations. *p < 0.005.

Collagen, sulfated glycosaminoglycan (sGAG), and hyaluronic acid (HA) content were analyzed before and after VAD (Fig. 2). VAD increased collagen from 0.12 ± 0.03 mg/mg dry weight in naïve fUB to 0.36 mg/mg dry weight in fUB-ECM (Fig. 2a). This increase is similar to reports on the decellularization of adult UB-ECM by agitation and likely due to collagen’s low solubility in water and the loss of more soluble cellular factors^37,38^. In contrast to collagen, VAD decreased both sGAG from 28 ± 1.3 µg/mg dry weight in naïve fUB to 18 ± 0.2 µg/mg dry weight in fUB-ECM (Fig. 2b) and HA from 29 µg/mg in naïve fUB to 14.7 µg/mg in fUB-ECM (Fig. 2c).

### FUB-ECM sheet morphology and tensile strength

**Figure 3:**
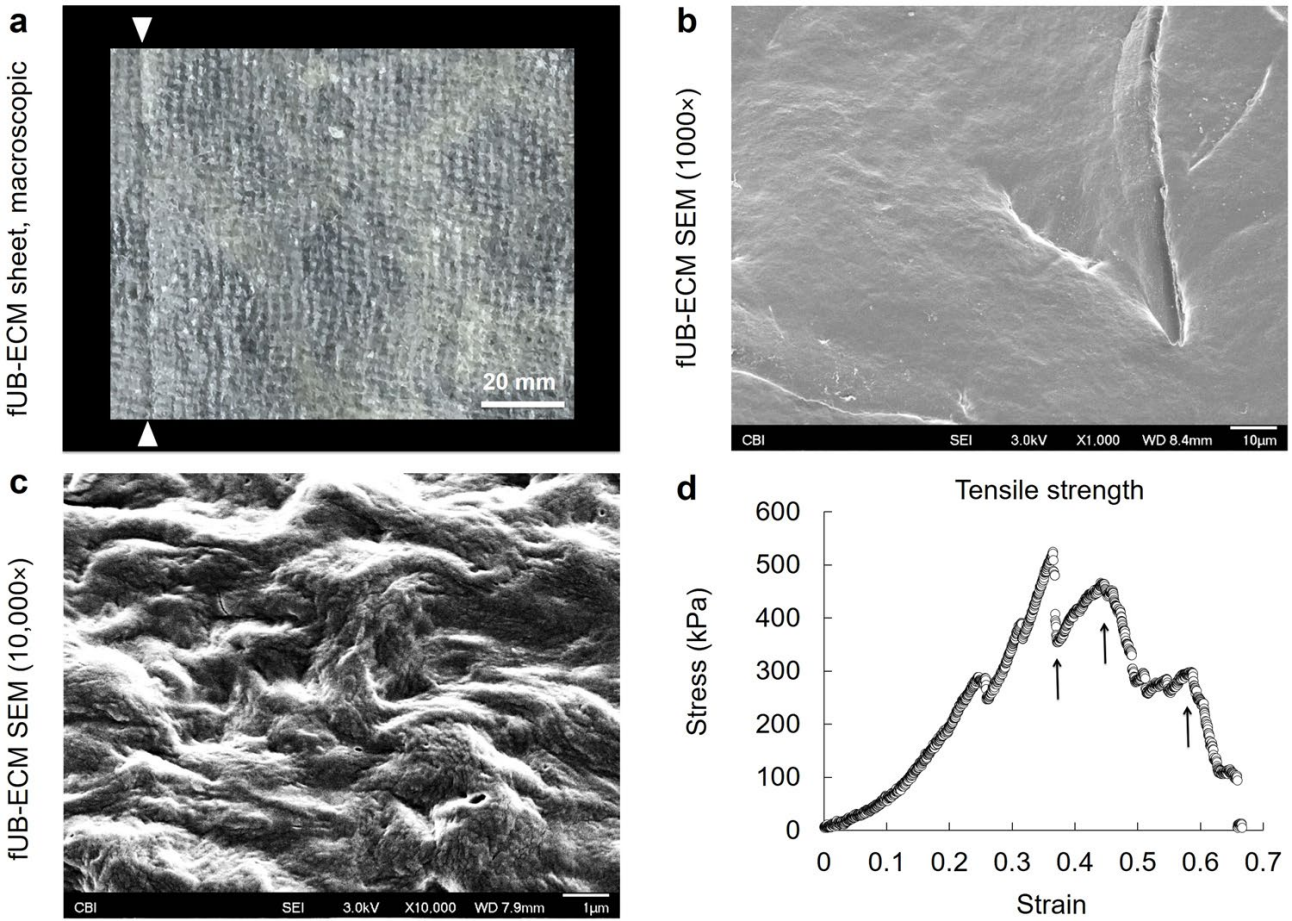
Single layer fUB-ECM vacuum pressed sheets are semi-transparent with good tensile strength. (**a**) Macroscopic view of a single layer, vacuum pressed fUB-ECM sheet showing a visible overlay, approximately 2–3 mm wide (arrowheads). (**b**,**c**) Scanning electron microscopy showed the the luminal, basement membrane, side of fUB-ECM sheets is relatively smooth at (**b**) 1000× with obvious collagen fibrils visible at (**c**) 10,000×. (**d**) Representative stress-strain curve shows vacuum-pressed fUB-ECM sheets have good tensile strength and multiple re-strengthening phases (arrows).

Single layer, fUB-ECM sheets were made by vacuum forming^14,39^, visualized macroscopically and microscopically using scanning electron microscopy (SEM), and analyzed mechanically by measuring tensile strength (Fig. 3). Macroscopically, vacuum formed fUB-ECM sheets were flat and semi-translucent with visible indentations from the metal grid used in the vacuum press (Fig. 3a). SEM showed the luminal, basement membrane surface of fUB-ECM was generally smooth at 1000× (Fig. 3b) with micrometer-scale collagen fibrils visible at 10,000× (Fig. 3c), similar to adult UB-ECM sheets^40^. Tensile strength testing showed fUB-ECM sheets had a Young’s modulus (strain < 1%) equal to 242 ± 4 kPa and a yield strength equal to 256 ± 7 kPa. The three re-strengthen stages in the stress-strain curve show the mechanical strength of fUB-ECM remains high even after partial damage (Fig. 3d).

### ION cut-repair model

**Figure 4:**
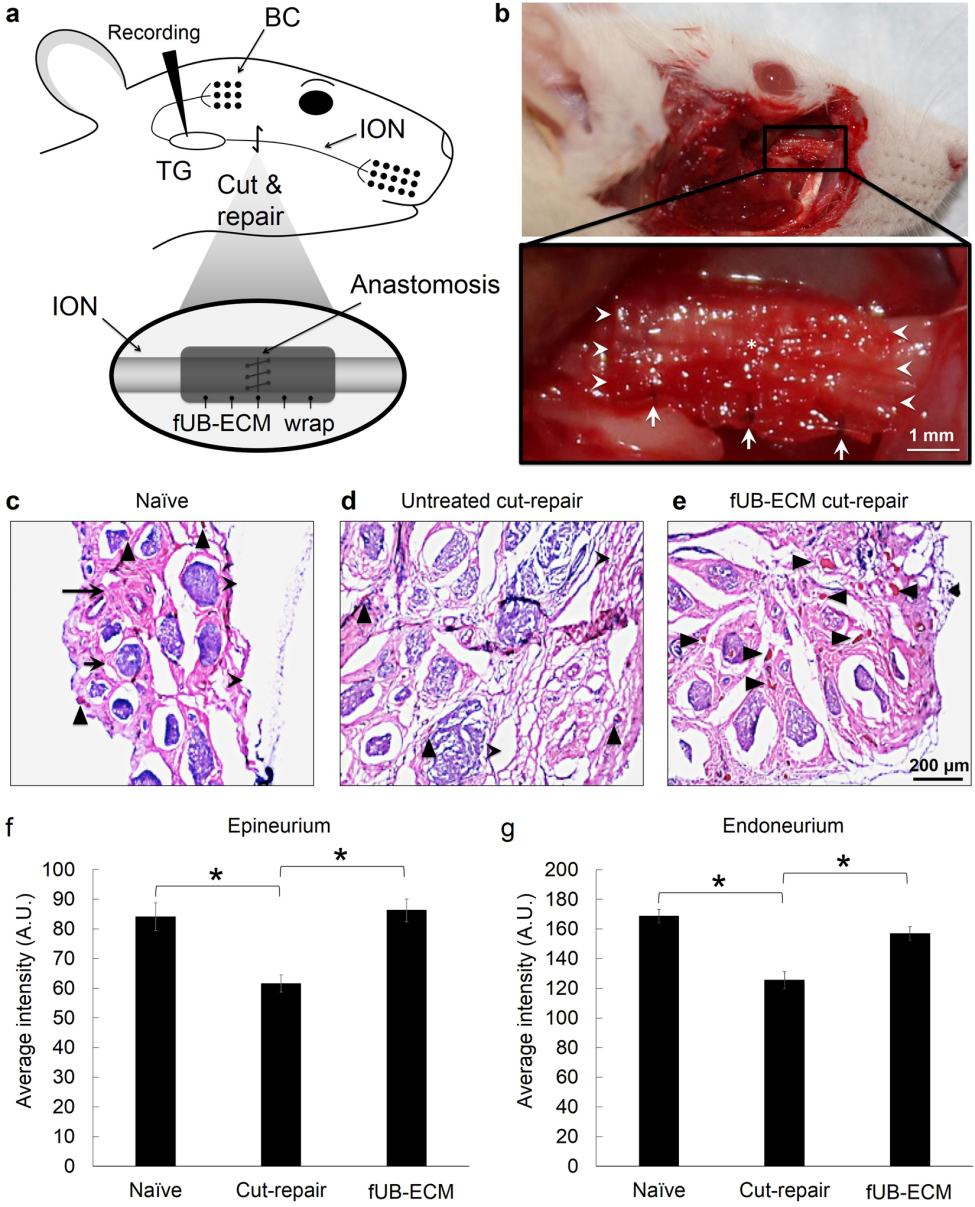
FUB-ECM positively modulates tissue remodeling after infraorbital nerve (ION) cut and repair. (**a**) Schematic of the ION cut and repair model showing the sutured cut and repair site (Anastomosis) and the fUB-ECM nerve wrap positioning around the anastomosis. Whisker-evoked response properties were recorded from trigeminal ganglion (TG) neurons innervating the whisker sinus via the ION branch of the trigeminal nerve. (**b**) After 28 days, fUB-ECM nerve wraps were intact and remained sutured around the ION. Higher magnification shows tissue growth in and around the fUB-ECM nerve wrap. Arrows indicate the sutures, arrowheads indicate the edges of the fUB-ECM nerve wrap, and the asterisk indicates the healed anastomosis. (**c**) H&E staining showed epineurial collagen (long arrows) and endoneurial nerve fascicles (dark purple, short arrows) in naïve IONs. Arrowheads indicate blood vessels of the vasa nervorum. (**d**) In untreated cut and repair IONs (Untreated cut-repair), both epineurial collagen and endoneurial nerve fascicles appeared to be loosely organized and fragmented. (**e**) In fUB-ECM wrapped cut and repair (fUB-ECM cut-repair) IONs, both the epi- and endoneurial organization more closely resembled naïve IONs. FUB-ECM cut-repair IONs also had larger and more numerous blood vessels (arrowheads), compared to both naïve and untreated cut-repair IONs. (**f**–**g**) Quantitatively, both (**f**) epineurial and (**g**) endoneurial densitometries were similar in naïve and fUB-ECM wrapped cut and repair (fUB-ECM) IONs, compared to untreated cut and repair (cut-repair) IONs. Data represent n ≥ 20 from at least 4 IONs per group. *p < 0.001.

To analyze fUB-ECM biocompatibility and bioactivity *in vivo*, we used an established ION cut-repair model in rat that has low variability and high analytic discrimination of functional mechanoreceptor reinnervation in the whisker sinus. The ION is a flat, purely sensory nerve that innervates and relays whisker-evoked responses from the whisker sinus to the barrel cortex in response to stimuli from two distinct mechanoreceptors, slowly adapting (SA) Merkel and rapidly adapting (RA) lanceolate receptors^41,42^. Whiskers are easily identified and ION axons show a gross topographical organization in the trigeminal ganglion, which is lost in the cut-repair model. The ION is easily accessed and reproducibly transected proximal to the whisker pad. After transection, the proximal and distal nerve trunks are easily placed in their correct anatomical orientation and repaired by direct end-to-end anastomosis by suturing the proximal and distal epineuria together without a gap defect^43^. In this study, the nerve was either left untreated after repair or a single layer fUB-ECM sheet was wrapped once around the repair site and sutured to itself securely without visibly compressing the nerve (Fig. 4a) by surgeons specializing in microsurgical PN repair. Moreover, for these initial studies, *in vivo* analyses were done at 28-days since biocompatibility, bioactivity, and function can all be analyzed at a single time point. By 28 days, sutured epineurial anastomoses have healed and transected ION axons have regrown and reinnervated whisker sinus mechanoreceptors sufficiently to analyze the initial whisker-evoked response properties electrophysiologically in the trigeminal ganglion.

### FUB-ECM nerve wraps increase positive tissue remodeling

At 28-days post cut-repair, the ION was exposed and examined. All nerves appeared healthy across all three groups without overt signs of infection, inflammation, or necrosis. FUB-ECM nerve wraps remained sutured in place, exhibiting healthy vascularized connective tissue growth in and around the wrap (Fig. 4b), without visible adhesions between the nerve wrap and the adjacent tissue bed. Compared to naïve IONs (Fig. 4c), histology showed ION tissue just anterior to the anastomosis site had distinct qualitatively differences in organization, depending on the post-injury treatment. In cut-repair animals, both the epineurium and the endoneurium showed signs of disorganization. The epineurium was loosely organized compared to the more densely organized epineurial collagen network seen in the naïve ION. Moreover, the endoneurium was less dense and the connective tissue appeared to be more randomly organized compared to naïve nerves (Fig. 4d). In contrast, fUB-ECM treated nerves had epi- and endoneurium organizations that closely resembled naïve IONs (Fig. 4e). Quantitatively, the density of the epi- and endoneurium was significantly reduced in the cut-repair nerves, whereas fUB-ECM wrapped cut-repair nerves were quantitatively similar to naïve, uninjured IONs (Fig. 4f,g).

### FUB-ECM nerve wraps increased neovascularization

FUB-ECM nerve wraps increased neovascularization significantly (Fig. 4c–e), consistent with ECM bioscaffolds in other nervous^44^ and non-nervous system tissues^15,25,26,31^. Quantitatively, all fUB-ECM wrapped ION sections showed more numerous and larger blood vessels that were approximately 271 ± 49% more frequent and approximately 304% larger (31 ± 12 a.u.) than vessels in naïve nerves, which averaged 10.2 ± 1.6 a.u., and approximately 475 ± 57% more frequent and approximately 525% larger than vessels in cut-repair nerves, which averaged 7.3 ± 1.9 a.u.

### FUB-ECM increased GAP-43 expression

**Figure 5:**
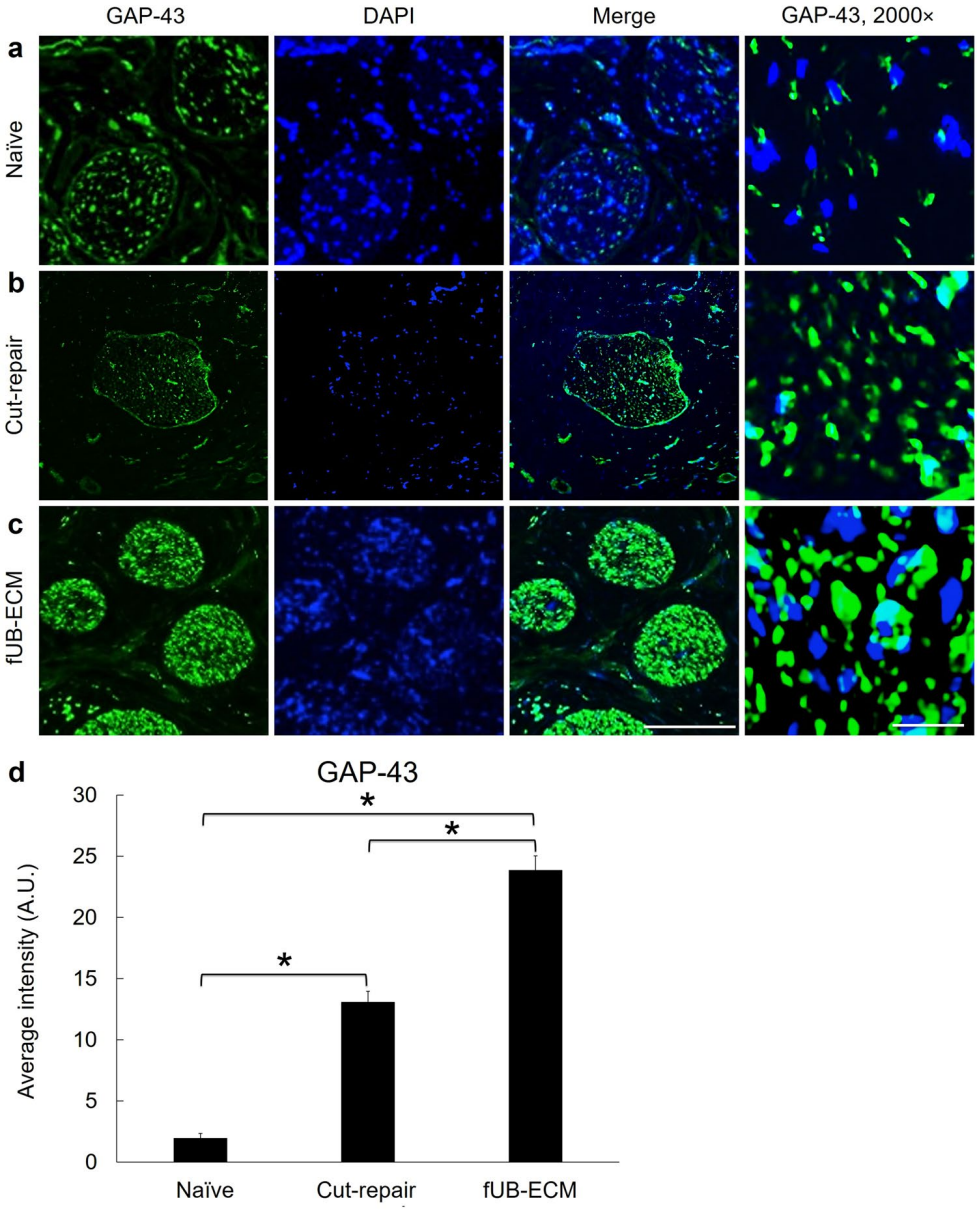
After ION cut and repair, fUB-ECM nerve wraps increase growth associated protein-43 (GAP-43) expression. Transverse sections anterior to the anastomosis site show GAP-43 immunoreactivity (green) and DAPI nuclear staining (blue) in (**a**) naïve, (**b**) cut and repair (cut-repair), and (**c**) fUB-ECM wrapped cut and repair (fUB-ECM) IONs. Higher magnification images (GAP-43, 2000×) show GAP-43 positive foci and DAPI positive nuclei. (**d**) Compared to both naïve and untreated cut and repair (cut-repair) IONs, GAP-43 immunoreactivity was greater in fUB-ECM wrapped cut and repair (fUB-ECM) IONs. Data represent n ≥ 20 from at least 4 IONs per group. *p < 0.001.

To determine if fUB-ECM nerve wraps can modulate axon growth signaling, GAP-43 immunoreactivity was analyzed (Fig. 5). GAP-43 immunofluorescence increased significantly in fUB-ECM wrapped IONs, approximately 12.5 times greater than in naïve and 185% greater than in untreated cut-repair IONs (Fig. 5a–d). Analysis of higher magnification images showed GAP-43 positive loci were larger in fUB-ECM treated IONs, averaging 13.2 ± 0.7 a.u., compared to 7.2 ± 1.3 a.u. in naïve and 9.5 ± 0.6 a.u. in untreated cut-repair IONs. Moreover, GAP-43 positive loci were more dense in fUB-ECM wrapped IONs, averaging 34.4 ± 3.1 a.u., compared to 4.2 ± 0.8 a.u. in naïve and 20.1 ± 1.8 in untreated cut-repair IONs.

### Neurofilament and myelin immunoreactivity

**Figure 6:**
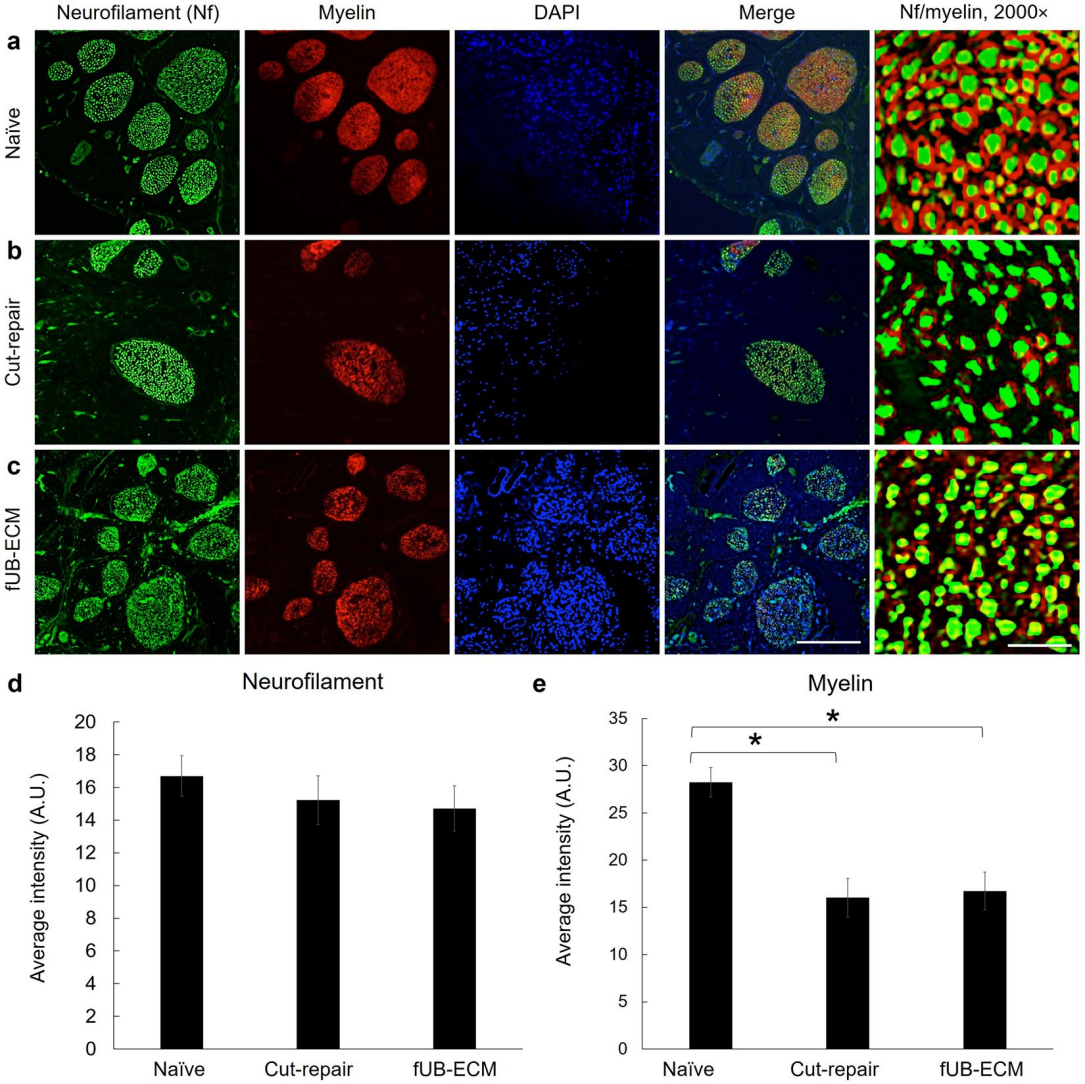
After ION cut and repair, neurofilament and myelin expression were unaltered by fUB-ECM nerve wraps. Transverse sections anterior to the anastomosis site show neurofilament (Nf, green) and myelin (red) immunoreactivity and DAPI nuclear staining (blue) in (**a**) naïve, (**b**) cut and repair (cut-repair), and (**c**) fUB-ECM wrapped cut and repair (fUB-ECM) IONs. Higher magnification images (Nf/myelin, 2000×) show myelinated neurofilament positive axons. (**d**) Quantitatively, neurofilament immunoreactivity was similar in all three groups. (**e**) Compared to naïve, myelin immunoreactivity was approximately 2-fold lower both in untreated cut and repair (cut-repair) and in fUB-ECM wrapped cut and repair (fUB-ECM) IONs. Data represent n ≥ 20 from at least 4 IONs per group. *p < 0.001.

To determine if fUB-ECM can modulate ION axon regrowth across the repair site and remyelination, neurofilament and myelin immunofluorescence were analyzed (Fig. 6). Neurofilament immunofluorescence was similar in all three groups (Fig. 6a–d), whereas myelin immunofluorescence was over 50% less in both untreated and fUB-ECM treated cut-repair groups compared to myelin immunofluorescence in naïve IONs (Fig. 6e). In higher magnification images (Fig. 6a–c, column 5), the density of neurofilament positive loci was similar across all three groups; naïve IONs averaged 30.50 ± 2.51 a.u, untreated cut-repair averaged 22.69 ± 1.69 a.u., and fUB-ECM averaged 26.79 ± 2.37 a.u. However, the area of neurofilament positive loci were greater in naïve IONs, averaging 28.54 ± 1.10 a.u., compared to 21.74 ± 0.82 a.u. in untreated cut-repair, and 22.42 ± 0.84 a.u in fUB-ECM cut-repair IONs. Analysis of higher magnification images showed that neurofilament positive axons were more myelinated, averaging 1.3 ± 0.10 a.u. per axon, compared to 0.70 ± 0.04 a.u. per axon in untreated cut-repair and 0.85 ± 0.07 a.u. per axon in fUB-ECM cut-repair IONs. Thus, most axons appear to regrow across the injury site in both untreated and fUBM-ECM cut-repair IONs. However, regrown axons are smaller and less myelinated in both groups compared to naïve ION axons.

**Figure 7:**
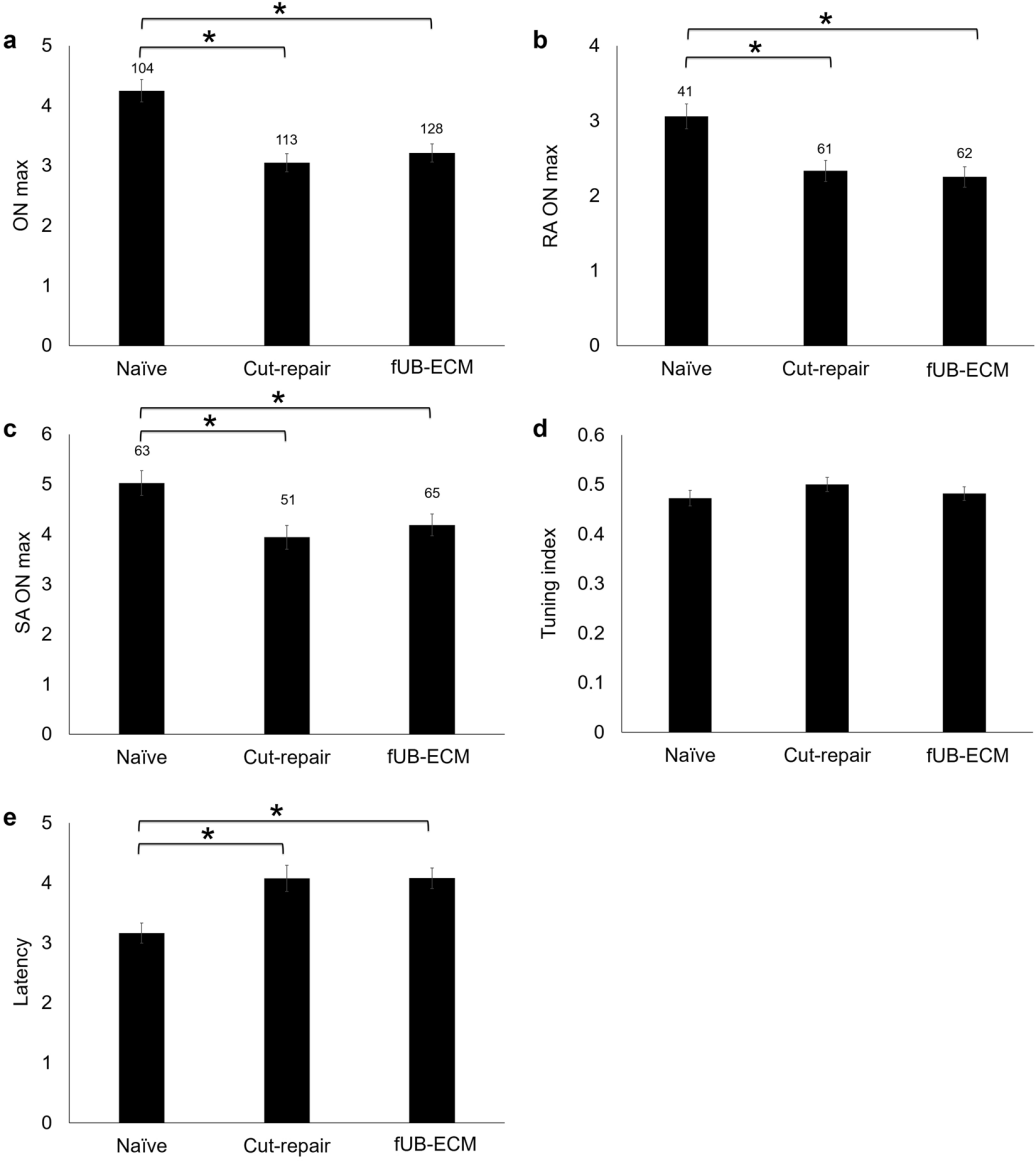
At 28 days post injury, the whisker-evoked response properties in ION cut-repair axons were unaltered by fUB-ECM nerve wraps. Response properties were recorded extracellularly from single units in the trigeminal ganglion. Whiskers activating the recording unit were identified using an auditory probe. Once identified, the whisker was controllably deflected to determine the maximum response angle and the type of mechanoreceptor activated, rapidly adapting (RA) lanceolate or slowly adapting (SA) Merkel, based on whether the plateau response exceeded the spontaneous firing activity (see methods). Compared to the response properties of naïve ION axons, the (**a**) maximum angular response (ON_max_), (**b**) RA ON_max_, and (**c**) SA ON_max_ were similarly lower in both untreated cut and repair (cut-repair) and fUB-ECM wrapped cut and repair (fUB-ECM) IONs. (**d**) The tuning index was similar across all three groups. (**e**) Compared to naïve, the latency was similarly greater both in untreated cut and repair (cut-repair) and in fUB-ECM wrapped cut and repair (fUB-ECM) IONs. The numbers above each bar in (**a**) indicate the total number of single-unit extracellular recordings per group. Each single-unit recording was then classified as either (**b**) RA (lanceolate) or (**c**) SA (Merkel), indicated by the numbers above each bar in graphs (**b**,**c**). Data represent at least 104 single-unit recordings from at least n = 8 animals per group. *p < 0.05.

Finally, to determine if fUB-ECM nerve wraps altered functional reinnervation, five whisker-evoked electrophysiological response parameters were analyzed from single unit ION cortical inputs (Fig. 7). The maximum stimulus onset (ON_max_) was defined as the spike count recorded over 20-ms, 1-ms after whisker deflection at the angle that initiated the greatest spike count. Expectedly, naïve ION axons had a greater ON_max_ than both untreated and fUB-ECM treated cut-repair ION axons (Fig. 7a). Next, at each cell’s maximally effective deflection angle, the ON_max_ responses were determined for either rapidly adapting (RA; Fig. 7b) lanceolate or slowly adapting (SA; Fig. 7c) Merkel mechanoreceptors. Consistent with previous findings^45^, RA units had smaller ON_max_ responses than SA units for all three experimental groups. Similar to ON_max_, both the RA ON_max_ (Fig. 7b) and the SA ON_max_ (Fig. 7c) for both untreated and fUB-ECM treated cut-repair groups were similarly lower than naïve. Angular tuning was quantified by comparing the responses recorded at the maximum effective angle with the averaged responses recorded from the other seven deflection angles (see Methods, Fig. 7d). In contrast to our previous findings^43^, the tuning index was similar across all three groups. Finally, compared to naïve, the latency was greater in both untreated and fUB-ECM cut-repair groups. Thus, fUB-ECM does not alter the whisker-evoked response properties of ION axons reinnervating the whisker sinus at 28 days post cut-repair.

## Discussion

Fetal UBs were successfully decellularized using a novel VAD chamber^33^ that can decellularize delicate tissues, including fetal tissues not possible using harsher agitation methods^19^. Decellularization protocols are typically designed to maximize the removal of DNA and other cellular contents while preserving the biologic and mechanical integrity of the ECM^46^. After VAD, H&E staining showed fUB-ECM retained eosin positive loci not typically seen in adult UB-ECM prepared by agitation^37^. However, eosin positive loci were hematoxylin negative, suggesting nuclear DNA was largely removed, and the eosin positive loci represented nuclear-associated, acidophilic proteins^47^ that remained bound indirectly to the ECM^48^. Though additional studies are needed, these results are consistent with VAD preserving more delicate fUB-ECM protein structures.

In fUB-ECM, dsDNA was higher compared to adult UB-ECM decellularized by agitation^14^, suggesting nucleic acids remained protected within fUB-ECMs. Though nucleic acid removal is desirable for clinical applications, variability exists in nucleic acid removal and detection, depending on the decellularization and quantification methods used^37,49^. Moreover, commercially available ECM scaffolds generally contain residual nucleic acids, including DNA^50,51^ and RNA^52,53^. Nucleic acids, including dsDNA, have been detected in numerous extracellular vesicle (EV) subtypes^54^ and at least some EV subtypes, like recently described matrix bound vesicles (MBV), resist decellularization, protect nucleic acids from enzymatic degradation, and are found in several commercial and laboratory generated ECM bioscaffolds^53^. Thus, further study is needed on the location, identity, and bioactivity of ECM-based nucleic acids.

FUB-ECM sheets have desirable mechanical and biocompatibility properties for use as off-the-shelf PN nerve wraps. Single layer, vacuum formed fUB-ECM sheets maintained their integrity after lyophilization, ETO sterilization, prolonged (weeks) storage at room temperature, and rehydration. After rehydration, the Young’s modulus was comparable to PEUU-UBM polymeric biohybrid ECM sheets^30^ and within the range of adult small intestine submucosa (SIS) ECM^55^, both successfully used in pre-clinical and clinical tissue repair studies^25,56,57^. *In vivo*, fUB-ECM nerve wraps maintained their integrity for 28-days, showed good suture retention, without obvious tears or other damage, and supported vascularized tissue growth over and within the wrap without overt signs of infection, inflammation, necrosis, or adhesions between the nerve wrap and the adjacent tissue bed. Since epineurial repair sites typically take about two weeks to heal, these results suggest fUB-ECM sheets may provide a naturally derived alternative material for protecting epineurial repair sites during healing. Moreover, since fUB-ECM is primarily composed of collagen fibrils, fUB-ECM may be a good candidate for repair by photochemical sealing^58^, which can reduce sutures and suture-related scarring and comorbidities.

FUB-ECM nerve wraps are bioactive; fUB-ECM nerve wraps positively modulated epi- and endoneurial tissue remodeling, including neovascularization, at ION repair sites. The ION was transected completely in this study, traversing the epineurium, perineurium, endoneurium, and importantly the vasa nervorum, which provides the blood supply to all PN tissues. Thus, transection of the vasa nervorum leads to both oxygen and metabolic deficiencies in affected tissues. Though the exact mechanisms between neovascularization and PN tissue remodeling are incompletely understood^59^, PN remodeling is closely linked to^60^ and differentially regulated by the degree of neovascularization^61,62^. Thus, revascularizing injured tissues is hypothesized to be a critical and rate limiting factor in constructive tissue remodeling. ECM bioscaffolds have been shown to increase neovascularization in other organs and tissues and these increases have been linked to more positive tissue remodeling and functional outcomes in bladder^25^, muscle^63^, tendon^64^ and nervous system tissues^44^, among others. Though injury to the native ECM can contribute to pathological angiogenesis^65^, the improved epi- and endoneurial organization, increased neovascularization, and functional data seen in fUB-ECM treated IONs are consistent with the hypothesis that, either directly or indirectly, fUB-ECM derived factors positively modulate PN tissue remodeling by increasing neovascularization.

The molecular and cellular mechanisms regulating neovascularization and axon regrowth after PN injury are poorly understood. However, recent studies suggest angiogenesis and neurogenesis are closely linked^66^ and likely modulated by the innate immune response to PN injury^67^. Macrophages are hypothesized to respond to and direct endothelial cell migration and the formation of new blood vessels in hypoxic tissues. In turn, newly formed blood vessels have been shown to guide Schwann cells and hence axon regrowth across PN injuries^68,69^. In support of these observations, alternatively activated, anti-inflammatory (M2-like) macrophages are linked to improved PN remodeling and positive outcomes^70^ and anti-inflammatory signaling can increase Schwann cell differentiation and migration^71^ and axon growth^67^. Moreover, PN-specific ECM bioscaffolds can increase M2-like macrophage polarization and Schwann cell migration^72^. After implantation, ECM bioscaffolds are rapidly invaded by macrophages, among other immune cells. Infiltrating macrophages degrade ECM bioscaffolds proteolytically^73^, releasing various bioactive matricryptic peptide fragments^19^ that can positively modulate the healing response by decreasing inflammatory signaling^74^, and increasing both angiogenesis^57^ and neurogenesis^28^. Whether ECM or macrophage derived factors or macrophages or a combination of the two enter epineurial repair sites to modulate inflammation and tissue repair is unknown. However, recent studies have shown that nerve wraps can release bioactive factors directly into wrapped nerves that can positively modulate both peripheral and central nerve regeneration^12,75^. Moreover, M2-like macrophages release a number of anti-inflammatory cytokines and other factors like extracellular EVs^76^ that can presumably diffuse into the wound site to positively modulate the phenotypes of both resident and other infiltrating immune cells^77^. Although additional studies are required, these results support the hypothesis that fUB-ECM derived factors modulate the innate immune response to induce alternatively activated (M2-like) macrophages^78^ and testing this hypothesis is an ongoing area of investigation^23^.

GAP-43 expression was increased significantly in fUB-ECM wrapped nerves. Both the area and density of GAP-43 positive foci were significantly greater in fUB-ECM wrapped nerves, suggesting fUB-ECM nerve wraps can modulate GAP-43 expression and possibly axon growth^79^. The increases in GAP-43 may indicate increased axon growth rate^80,81^ or increased axonal branching from nodes of Ranvier^82.^ However, increased GAP-43 is unlikely to reflect an increase in the number of axons crossing the repair site since similar numbers of neurofilament positive axons were detected in both untreated and fUB-ECM treated cut and repair nerves and these numbers were similar to the number of axons in naïve nerves. Thus, most transected ION axons appear to have regrown across the injury site at 28 days post injury. Whether GAP-43 was expressed above some threshold or whether the rate of axon regrowth was greater in fUB-ECM treated nerves is currently under investigation. Adult UB-ECM derived factors can increase axon growth rate^29^, neurogenesis in the PNS^83^, and Schwann cell migration and differentiation^72^. Nerve growth factor (NGF), produced by Schwann cells, stimulates and guides peripheral nerve axons and NGF can regulate GAP-43 mRNA stability and translation^84,85^. Thus, the observation that fUB-ECM can influence GAP-43 expression, opens up a number of experimental approaches to understanding the mechanisms regulating GAP-43 expression and peripheral nerve axon regrowth after injury.

In this study, positive tissue remodeling at epineurial repair sites did not predict functional improvements at 28-days. The ION cut and repair model provides several advantages for analyzing functional axonal reinnervation. The ION relays sensory stimuli from the whisker sinus to the barrel cortex. Whiskers are easily identified, mechanically deflected in a controlled manner to activate two distinct classes of mechanoreceptors, the slowly adapting (SA) Merkel and the rapidly adapting (RA) lanceolate receptors^86–88^. Since most ION axons have little to no spontaneous activity in rats, clean and stable, extracellular single-unit recordings can be acquired from both SA and RA receptors^89,90^. In this study, the response properties of both SA and RA mechanoreceptor subtypes were similar in untreated and fUB-ECM treated cut and repair animals after 28 days, indicating that positive tissue remodeling alone does not predict return of function. Alternatively, the data suggest myelination is limiting. Compared to naïve IONs, myelination was reduced similarly by over 50% in both untreated and fUB-ECM cut-repair IONs. In ongoing studies, longitudinal sections are being analyzed at different time points to evaluate Schwann cell migration^69^, myelin thickness, organization, and nodes of Ranvier^91,92^. Moreover, although the response properties of individual ganglion cells cannot be extrapolated reliably to determine ION nerve conduction of the ION^43^, nerve conduction can be analyzed by directly stimulating the ION and used in conjunction with histology in longitudinal sections to determine if remyelination in untreated and fUB-ECM cut-repair IONs correlates with ION nerve conduction rates.

With respect to the functional data, a number of caveats are worth discussing. After ION transection and mechanoreceptor denervation, all lanceolates generally degenerate^35^, whereas Merkel cells typically do not^93^. After whisker sinus reinnervation, lanceolate receptors do regenerate. However, whisker sinus can remain altered months after reinnervation in ION cut and repair animals^43,94^. Lanceolate receptors (RA units) require 11–16 weeks to reach control levels, whereas, although Merkel receptors (SA units) do not typically degenerate, Merkel-evoked stimuli can take 8–12 weeks to reach control levels^43^. Thus, although whisker-evoked responses are analyzable at 28 days, these initial functional studies were designed primarily to assess material biocompatibility and increases in the degree or the rate of functional reinnervation may be undetectable. As such, the data from this study argues that fUB-ECM nerve wraps should be studied over an expanded range of time points. Moreover, trigeminal ganglion cells axons, making up the ION, exhibit a coarse topographical organization with respect to the whiskers^45,95^, which is lost in transected animals^43^, likely due to axonal misrouting and negative effects on CNS spatial mapping^96^ and spike precision^97,98^, factors necessary for accurate somatosensory discrimination in tissues like the whisker pads^42^. Thus, studies on ION axon guidance and appropriate topographical reinnervation are also warranted. Finally, ION transection typically leads to uneven mechanoreceptor degeneration within the sinus hair follicle and an increase in the tuning index following reinnervation^45,99^. However, we failed to detect a difference in the tuning index at 28 days. Thus, additional studies are required to determine the rate of mechanoreceptor regeneration and effects of reinnervation on mechanoreceptor maturation.

## Conclusion

ECM nerve wraps, derived from healthy pro-regenerative tissues, may provide distinct experimental and potentially clinical advantages over current clinically used PN wraps. This study showed fUB-ECM nerve wraps are safe biocompatible and bioactive devices and suggest additional time points and electrophysiological and histological analyses are not only warranted but also likely to reveal new biology and therapeutic targets that can positively modulate the default healing response in the PNS. Though speculative, with improved remodeling within the ION, reduced inflammation, and the preservation or restoration of appropriate topographic nerve reinnervation, ECM treatment either alone or augmented with other factors that increase the rate of remyelination^100^, for instance, may be able to promote more successful return of function. Moreover, FUB-ECM is a tunable platform that can be engineered mechanically and biochemically to deliver specific factors with temporal and spatial control^30,75,101^, based on the nature and the scope of the injury.

## Methods

### Animal use statement

Animal research protocols followed the National Institutes of Health guidelines for animal care and were approved by the University of Pittsburgh Institutional Animal Care and Use Committee.

### Animals, reagents and tissues

Lewis rats (8–10 weeks, Charles River, Wilmington, MA) were used for *in vivo* experiments. Chemicals were supplied by Sigma-Aldrich (St. Louis, MO) and cell culture reagents by Life Technologies (San Diego, CA) unless specified. Frozen fetal pigs (5–7 inches) were supplied by Nebraska Scientific (Omaha, NE) and adult market weight pig bladders by Thoma’s Meat market (Saxonburg, PA).

### Fetal UB-ECM

Frozen fetal pigs were thawed at 4 °C, the urinary bladders removed, and the connective and adipose tissues dissected from the serosal surface. The tunica serosa, tunica submucosa, and majority of the tunica muscularis mucosa were mechanically removed, leaving the basement membrane and the tunica propria intact as described^102^. Fetal urinary bladders were decellularized using three sequential washes in a VAD chamber: nanopure water, 3% triton X-100, and 3 M NaCl as described^29^. During each wash, the pressure was cycled between ambient and 0 psi 30× for 1 min per cycle by code custom written in LabView (National Instruments, Austin, TX). The three washes were repeated eight times. After VAD, the resulting fUB-ECM bioscaffolds were incubated with deoxyribonuclease (DNAase, 300 Kunitz units per ml, Sigma) for 1 hr, 0.1% peracetic acid (Rochester Midland Corp., Rochester, NY) in ethanol (4%) for 1 hr, and then washed in PBS followed by nanopure water 3× for 15 min each.

### FUB-ECM sheets

Single layer fUB-ECM sheets were made by vacuum pressing (Model D4B, Leybold, Export, PA,) as described^14,39^. Briefly, 4–5 fUB-ECM strips (approx. 1 × 2 cm) were flattened on a metal mesh plate with each fUB-ECM strip slightly overlapped, luminal side up. A second metal plate was placed on top and a constant vacuum of 85 kPa was applied for 5 h to dehydrate the sheet. The dehydrated fUB-ECM sheet was sterilized with ethylene oxide (ETO) as described^103^ (EOGas Sterilizer Series 3+, Andersen Products, NC), and stored sealed at room temperature. Before use, fUB-ECM sheets were rehydrated in PBS at 4 °C overnight.

### DNA analysis

Qualitatively, residual DNA was visualized by fixing non-lyophilized fUB-ECM scaffolds in 10% neutral buffered formalin. Fixed fUB-ECM was embedded in paraffin, sectioned, and stained with hematoxylin and eosin (H&E). Quantitatively, DNA content and base pair length were analyzed as described^52^. Briefly, fUB or fUB-ECM was digested with 0.1 mg/ml proteinase K at 50 °C in Tris buffer (10 mM Tris-HCl, pH 8.0, 100 mM NaCl, 25 mM EDTA) overnight. Protein was removed by phenol/chloroform extraction and centrifugation (10,000 g). The aqueous phase was mixed with 3 M sodium acetate, 100% ethanol and centrifuged. The DNA pellet was rinsed with 70% ethanol, centrifuged, and air-dried. Residual DNA length was determined by gel electrophoresis using 1.0% agarose gels with ethidium bromide (2 hr at 60 V) followed by imaging with an ultraviolet transilluminator (ChemiDoc Touch Imager, Bio-Rad, CA). Double-stranded DNA was quantified using PicoGreen according to the manufacturer’s instructions (P7589, Invitrogen, Carlsbad, CA).

### Collagen, sGAG, and HA quantification

In naïve fUB or fUB-ECM, collagen content was measured using a sircol assay kit (S1000; Biocolor Ltd., UK) and sGAG was measured using a Blyscan sGAG assay kit (B1000, Biocolor Ltd., UK) according to the manufacture’s instructions. Briefly, fUB or fUB-ECM (50 mg/ml) was digested with proteinase K, as described above, and collagen measured by absorption at 550 nm and sGAG by absorption at 656 nm using a SpectraMax M2 spectrophotometer (Molecular Devices, LLC. Sunnyvale, CA). HA content was measured in neutralized, pepsin solubilized fUB or fUB-ECM (1 mg/mL) by ELISA according to the manufacturer’s instructions (DHYAL0, R&D systems).

### Scanning electron microscopy

FUB-ECM sheets were examined by scanning electron microscopy (SEM) as described^104^. Briefly, fUB- ECM sheets were sputter-coated with gold/palladium alloy (4.5 nm) using a Sputter Coater 108auto (Cressington Scientific Instruments, UK) and imaged with a JEOL JSM6330f scanning electron microscope (JEOL, Peabody, MA).

### FUB-ECM tensile strength

The tensile strength of fUB-ECM sheets was measured with an ElectroForce 3200 Series II (Bose, Minnesota, US) equipped with a 1000 g mechanical load cell as described^30^. Briefly, the fUB-ECM sheets were re-hydrated in deionized water at 4 °C for 24 h. After hydration, the tensile stress-strain curve was measured immediately at a pulling speed of 0.1 mm/s. Three fUB-ECM sheets from three different fetal bladders and VADs were tested.

### Infraorbital nerve cut and repair

Surgeries were done as previously described^43^ and followed standard operating procedures for trigeminal nerve repair^105^. All surgeries were done by practicing plastic surgeons that specialize in microsurgical nerve repair and routinely use commercially available nerve wraps clinically. Briefly, the ION was exposed proximal to the mystacial (whisker) pad and distal to the infraorbital foramen and transected completely. The ION was immediately repaired by suturing the proximal and distal nerve trunk epineria together in their correct anatomical orientation and without a gap defect. After repair, the anastomosis was either left untreated or wrapped once with a 2 mm × 5 mm fUB-ECM sheet sutured to itself using size 6–0 plain gut sutures (Ethicon, NJ). Animal numbers were determined by power analysis^106^ using G*power software (G*power software 3.1.9.2, Germany). The effect size was calculated to be 0.6 based on our previous experiments. Power analysis for ANOVA dictated at least n = 4 animals per group for histological analysis and at least n = 5 animals per group for functional analysis to achieve 80% power for α = 0.05.

### Trigeminal ganglion cell electrophysiology and analysis

Electrophysiological recordings were done as previously described^43^ at 28 days post surgery. A single time point was chosen to minimize animal usage and to enable both biocompatibility and bioactivity to be sufficiently analyzed. At 28 days, mechanoreceptor regeneration is ongoing. However, transected trigeminal axons have regrown and reinnervated some whisker sinus sufficiently to analyze the initial whisker-evoked responses electrophysiologically. To expose the left trigeminal ganglion, a craniotomy was done and extracellular recordings of single trigeminal ganglion cells were acquired using tungsten microelectrodes controlled by a motorized microdrive. Action potentials were recorded using standard amplification and band-pass filtering (300 Hz-10 kHz). Single units were identified by spike waveforms that were collected at 32 kHz and analyzed using a custom spike-sorting software program (LabVIEW, National Instruments, Austin, TX). The principal whisker activating the recorded cell was first identified and then a computer controlled multi-angle piezoelectric stimulator^90^, was then attached to the base of the whisker and used to deflect the whisker in eight angular directions. Each whisker was deflected 80 times (10 deflections over 8 angles). Responses were classified as slowly adapting (SA) or rapidly adapting (RA) based on the magnitude of the plateau response with respect to the spontaneous firing rate (95% confidence limit, one-tail *t*-test). For functional analysis, at least 100 single unit recordings were acquired from at least eight animals per group as follows: naïve (n = 8, 104 units), untreated cut and repair (n = 10, 113 units), and fUB-ECM cut and repair (n = 9, 128 units). Data were analyzed as described^43^ and statistical significance between groups determined by one-way ANOVA with Tukey post hoc analysis, and a significance level of *p* < 0.05 for all tests. Error bars represent standard error of the mean (SEM).

### Immunohistochemistry (IHC)

Animals were euthanized 28 days after surgery and the trigeminal nerves dissected and fixed immediately with 4% paraformaldehyde in PBS for at least 2 hr. Fixed nerves were cryoprotected in 30% sucrose in PBS for 4 to 12 hr before embedding in optimal cutting temperature (OCT) medium (Tissue-Tek; Miles Inc, Elkhart, IN) and freezing in liquid nitrogen. Frozen nerves were stored at −80 °C before sectioning. Transverse sections (15 µm) of the ION were cut just anterior to the repair site on a cryostat (Leica CM 1850; Leica Biosystems, Wetzlar, Germany). ION sections were H&E stained or labeled with anti-neurofilament medium chain (1:400, ThermoScientific/Pierce), anti-myelin (1:300, Life Technologies), or anti-GAP 43 (1:300, Thermo Scientific) and nuclei were stained with DAPI. Fluorescence intensity was measured using ImageJ software as previously described^107^. Briefly, the image was opened in ImageJ. Using a selection tool, the outline of the fascicle was traced and the area, mean fluorescence, and integrated density were measured. Background measurements were acquired in a similar fashion. The corrected total fluorescence (CTCF) for each antibody was calculated using the formula: CTCF = integrated density − (area × mean background fluorescence). High magnification images were used to calculate neurofilament loci density, neurofilament area, and myelination. Images were opened in ImageJ and the diameters of neurofilament positive stains measured to determine neurofilament area. Neurofilament density was calculated as percentage neurofilament coverage per image according to the following formula: neurofilament density = (neurofilament positive stain area/total area of image) *100. Finally myelination was calculated as a ratio of myelination to neurofilament. Total area of myelin and neurofilament immunofluorescence were measured separately and expressed as a ratio of myelin/neurofilament. One-way ANOVA was used to determine significance (*p* < 0.05) between groups with n ≥ 4 animals per condition.

### Data availability

The datasets generated and analyzed in the current study are available from the corresponding author on reasonable request.

## Electronic supplementary material

DNA gel electrophoresis

## References

[1] Grinsell D, Keating CP (2014). Peripheral nerve reconstruction after injury: a review of clinical and experimental therapies. Biomed Res Int.

[2] Lundborg G, Rosen B (2007). Hand function after nerve repair. Acta Physiol (Oxf).

[3] Westling G, Johansson RS (1984). Factors influencing the force control during precision grip. Experimental brain research.

[4] Terzis J, Faibisoff B, Williams B (1975). The nerve gap: suture under tension vs. graft. Plast Reconstr Surg.

[5] Aldskogius H, Arvidsson J (1978). Nerve cell degeneration and death in the trigeminal ganglion of the adult rat following peripheral nerve transection. J Neurocytol.

[6] Kline DG, Hayes GJ (1964). The Use of a Resorbable Wrapper for Peripheral-Nerve Repair; Experimental Studies in Chimpanzees. J Neurosurg.

[7] Kokkalis ZT, Mavrogenis AF, Ballas EG, Papagelopoulos PJ, Soucacos PN (2015). Collagen nerve wrap for median nerve scarring. Orthopedics.

[8] Patel VR (2015). Dehydrated Human Amnion/Chorion Membrane Allograft Nerve Wrap Around the Prostatic Neurovascular Bundle Accelerates Early Return to Continence and Potency Following Robot-assisted Radical Prostatectomy: Propensity Score-matched Analysis. Eur Urol.

[9] Kim PD (2010). Collagen nerve protector in rat sciatic nerve repair: A morphometric and histological analysis. Microsurgery.

[10] Shintani, K. *et al*. Protective effect of biodegradable nerve conduit against peripheral nerve adhesion after neurolysis. *J Neurosurg*, 1–10, 10.3171/2017.4.JNS162522 (2017). 10.3171/2017.4.JNS162522 29053076

[11] Economides JM, DeFazio MV, Attinger CE, Barbour JR (2016). Prevention of Painful Neuroma and Phantom Limb Pain After Transfemoral Amputations Through Concomitant Nerve Coaptation and Collagen Nerve Wrapping. Neurosurgery.

[12] Suzuki K (2017). Electrospun nanofiber sheets incorporating methylcobalamin promote nerve regeneration and functional recovery in a rat sciatic nerve crush injury model. Acta Biomater.

[13] Rosen A, Tardast A, Shi TJ (2016). How Far Have We Come in the Field of Nerve Regeneration After Trigeminal Nerve Injury?. Curr Oral Health Rep.

[14] Badylak SF, Freytes DO, Gilbert TW (2009). Extracellular matrix as a biological scaffold material: Structure and function. Acta Biomater.

[15] Brown BN (2011). Extracellular matrix as an inductive template for temporomandibular joint meniscus reconstruction: a pilot study. J Oral Maxillofac Surg.

[16] Badylak SF, Dziki JL, Sicari BM, Ambrosio F, Boninger ML (2016). Mechanisms by which acellular biologic scaffolds promote functional skeletal muscle restoration. Biomaterials.

[17] Badylak SF (2005). Esophageal reconstruction with ECM and muscle tissue in a dog model. J Surg Res.

[18] Meng F, Modo M, Badylak SF (2014). Biologic scaffold for CNS repair. Regen Med.

[19] Ren, T., van der Merwe, Y. & Steketee, M. B. Developing Extracellular Matrix Technology to Treat Retinal or Optic Nerve Injury(1,2,3). *eNeuro* **2**, 10.1523/ENEURO.0077-15.2015 (2015). 10.1523/ENEURO.0077-15.2015 PMC4603254 26478910

[20] Kurtz A, Oh SJ (2012). Age related changes of the extracellular matrix and stem cell maintenance. Prev Med.

[21] Li J (2014). Rejuvenation of chondrogenic potential in a young stem cell microenvironment. Biomaterials.

[22] Dziki, J. L., Huleihel, L., Scarritt, M. E. & Badylak, S. F. Extracellular Matrix Bioscaffolds as Immunomodulatory Biomaterials. *Tissue Eng Part A*, 10.1089/ten.TEA.2016.0538 (2017). 10.1089/ten.tea.2016.0538 PMC6112165 28457179

[23] Huleihel, L. *et al*. Macrophage phenotype in response to ECM bioscaffolds. *Semin Immunol*, 10.1016/j.smim.2017.04.004 (2017). 10.1016/j.smim.2017.04.004 PMC5612880 28736160

[24] Remlinger NT (2013). Urinary bladder matrix promotes site appropriate tissue formation following right ventricle outflow tract repair. Organogenesis.

[25] Badylak SF, Kropp B, McPherson T, Liang H, Snyder PW (1998). Small intestinal submucosa: a rapidly resorbed bioscaffold for augmentation cystoplasty in a dog model. Tissue Eng.

[26] Fercana GR (2017). Perivascular extracellular matrix hydrogels mimic native matrix microarchitecture and promote angiogenesis via basic fibroblast growth factor. Biomaterials.

[27] Armstrong SJ, Wiberg M, Terenghi G, Kingham PJ (2007). ECM molecules mediate both Schwann cell proliferation and activation to enhance neurite outgrowth. Tissue Eng.

[28] Agrawal V, Brown BN, Beattie AJ, Gilbert TW, Badylak SF (2009). Evidence of innervation following extracellular matrix scaffold-mediated remodelling of muscular tissues. J Tissue Eng Regen Med.

[29] Faust A (2017). Urinary bladder extracellular matrix hydrogels and matrix-bound vesicles differentially regulate central nervous system neuron viability and axon growth and branching. J Biomater Appl.

[30] Hong Y (2011). Mechanical properties and *in vivo* behavior of a biodegradable synthetic polymer microfiber-extracellular matrix hydrogel biohybrid scaffold. Biomaterials.

[31] D’Amore, A. *et al*. Nitro-oleic acid (NO2OA) release enhances regional angiogenesis in a rat abdominal wall defect model. *Tissue Eng Part A*, 10.1089/ten.TEA.2017.0349 (2017). 10.1089/ten.tea.2017.0349 PMC5984564 29187125

[32] Saldin, L. T., Cramer, M. C., Velankar, S. S., White, L. J. & Badylak, S. F. Extracellular Matrix Hydrogels from Decellularized Tissues: Structure and Function. *Acta Biomater*, 10.1016/j.actbio.2016.11.068 (2016). 10.1016/j.actbio.2016.11.068 PMC5253110 27915024

[33] Gilbert, T. W., Hobson, C. M, Ungchusri, E. N. Method and apparatus for decellularization of tissue (WO 2015134618 A1) (2015).

[34] Renehan WE, Munger BL (1986). Degeneration and regeneration of peripheral nerve in the rat trigeminal system. II. Response to nerve lesions. J Comp Neurol.

[35] Renehan WE, Klein BG, Chiaia NL, Jacquin MF, Rhoades RW (1989). Physiological and anatomical consequences of infraorbital nerve transection in the trigeminal ganglion and trigeminal spinal tract of the adult rat. J Neurosci.

[36] Pali J, Negyessy L (2002). Reinnervation of a single vibrissa after nerve excision in the adult rat. Neuroreport.

[37] Rosario DJ (2008). Decellularization and sterilization of porcine urinary bladder matrix for tissue engineering in the lower urinary tract. Regen Med.

[38] Slivka PF (2014). Fractionation of an ECM hydrogel into structural and soluble components reveals distinctive roles in regulating macrophage behavior. Biomater Sci.

[39] Freytes DO (2005). Analytically derived material properties of multilaminated extracellular matrix devices using the ball-burst test. Biomaterials.

[40] Turner WS (2012). Cardiac tissue development for delivery of embryonic stem cell-derived endothelial and cardiac cells in natural matrices. J Biomed Mater Res B Appl Biomater.

[41] Petersen CC (2007). The functional organization of the barrel cortex. Neuron.

[42] Waite PM, de Permentier P (1991). The rat’s postero-orbital sinus hair: I. Brainstem projections and the effect of infraorbital nerve section at different ages. J Comp Neurol.

[43] Xiao B, Zanoun RR, Carvell GE, Simons DJ, Washington KM (2016). Response properties of whisker-associated primary afferent neurons following infraorbital nerve transection with microsurgical repair in adult rats. J Neurophysiol.

[44] Tukmachev D (2016). Injectable Extracellular Matrix Hydrogels as Scaffolds for Spinal Cord Injury Repair. Tissue Eng Part A.

[45] Lichtenstein SH, Carvell GE, Simons DJ (1990). Responses of rat trigeminal ganglion neurons to movements of vibrissae in different directions. Somatosens Mot Res.

[46] Gilbert TW, Sellaro TL, Badylak SF (2006). Decellularization of tissues and organs. Biomaterials.

[47] Fischer AH, Jacobson KA, Rose J, Zeller R (2008). Hematoxylin and eosin staining of tissue and cell sections. CSH Protoc.

[48] Wang N, Tytell JD, Ingber DE (2009). Mechanotransduction at a distance: mechanically coupling the extracellular matrix with the nucleus. Nat Rev Mol Cell Biol.

[49] Silva-Benitez E (2015). Quantification of DNA in urinary porcine bladder matrix using the ACTB gene. In Vitro Cell Dev Biol Anim.

[50] Zheng MH (2005). Porcine small intestine submucosa (SIS) is not an acellular collagenous matrix and contains porcine DNA: possible implications in human implantation. J Biomed Mater Res B Appl Biomater.

[51] Derwin KA, Baker AR, Spragg RK, Leigh DR, Iannotti JP (2006). Commercial extracellular matrix scaffolds for rotator cuff tendon repair. Biomechanical, biochemical, and cellular properties. J Bone Joint Surg Am.

[52] Gilbert TW, Freund JM, Badylak SF (2009). Quantification of DNA in biologic scaffold materials. J Surg Res.

[53] Huleihel L (2016). Matrix-bound nanovesicles within ECM bioscaffolds. Sci Adv.

[54] Yanez-Mo M (2015). Biological properties of extracellular vesicles and their physiological functions. J Extracell Vesicles.

[55] Obermiller JF, Hodde JP, McAlexander CS, Kokini K, Badylak SF (2004). A comparison of suture retention strengths for three biomaterials. Med Sci Monit.

[56] Boruch AV, Nieponice A, Qureshi IR, Gilbert TW, Badylak SF (2010). Constructive remodeling of biologic scaffolds is dependent on early exposure to physiologic bladder filling in a canine partial cystectomy model. J Surg Res.

[57] D’Amore A (2016). Bi-layered polyurethane - Extracellular matrix cardiac patch improves ischemic ventricular wall remodeling in a rat model. Biomaterials.

[58] Fairbairn NG (2016). Improving Outcomes in Immediate and Delayed Nerve Grafting of Peripheral Nerve Gaps Using Light-Activated Sealing of Neurorrhaphy Sites with Human Amnion Wraps. Plast Reconstr Surg.

[59] Nukada H, Dyck PJ (1986). Neovascularization after ischemic nerve injury. Exp Neurol.

[60] Nukada H (1988). Post-traumatic endoneurial neovascularization and nerve regeneration: a morphometric study. Brain Res.

[61] Kirchmair R (2007). Therapeutic angiogenesis inhibits or rescues chemotherapy-induced peripheral neuropathy: taxol- and thalidomide-induced injury of vasa nervorum is ameliorated by VEGF. Mol Ther.

[62] Pola R (2004). Age-dependent VEGF expression and intraneural neovascularization during regeneration of peripheral nerves. Neurobiol Aging.

[63] Zhang J (2016). Perfusion-decellularized skeletal muscle as a three-dimensional scaffold with a vascular network template. Biomaterials.

[64] Zantop T, Gilbert TW, Yoder MC, Badylak SF (2006). Extracellular matrix scaffolds are repopulated by bone marrow-derived cells in a mouse model of achilles tendon reconstruction. J Orthop Res.

[65] Neve A, Cantatore FP, Maruotti N, Corrado A, Ribatti D (2014). Extracellular matrix modulates angiogenesis in physiological and pathological conditions. Biomed Res Int.

[66] Wang H (2017). Overlapping Mechanisms of Peripheral Nerve Regeneration and Angiogenesis Following Sciatic Nerve Transection. Front Cell Neurosci.

[67] Mokarram N, Merchant A, Mukhatyar V, Patel G, Bellamkonda RV (2012). Effect of modulating macrophage phenotype on peripheral nerve repair. Biomaterials.

[68] Fantin A (2010). Tissue macrophages act as cellular chaperones for vascular anastomosis downstream of VEGF-mediated endothelial tip cell induction. Blood.

[69] Cattin AL (2015). Macrophage-Induced Blood Vessels Guide Schwann Cell-Mediated Regeneration of Peripheral Nerves. Cell.

[70] Enam SF (2017). Enrichment of endogenous fractalkine and anti-inflammatory cells via aptamer-functionalized hydrogels. Biomaterials.

[71] Clements MP (2017). The Wound Microenvironment Reprograms Schwann Cells to Invasive Mesenchymal-like Cells to Drive Peripheral Nerve Regeneration. Neuron.

[72] Prest, T. A. *et al*. Nerve-specific, xenogeneic extracellular matrix hydrogel promotes recovery following peripheral nerve injury. *J Biomed Mater Res A*, 10.1002/jbm.a.36235 (2017). 10.1002/jbm.a.36235 PMC5745279 28891122

[73] Valentin JE, Stewart-Akers AM, Gilbert TW, Badylak SF (2009). Macrophage participation in the degradation and remodeling of extracellular matrix scaffolds. Tissue Eng Part A.

[74] Swinehart, I. T. & Badylak, S. F. Extracellular matrix bioscaffolds in tissue remodeling and morphogenesis. *Dev Dyn*, 10.1002/dvdy.24379 (2015). 10.1002/dvdy.24379 PMC4755921 26699796

[75] Van der Merwe, Y. *et al*. An Elastomeric Polymer Matrix, PEUU-Tac, Delivers Bioactive Tacrolimus Transdurally to the CNS in Rat. *EBioMedicine*, 10.1016/j.ebiom.2017.11.017 (2017). 10.1016/j.ebiom.2017.11.017 PMC5832622 29208469

[76] van der Merwe Y, Faust AE, Steketee MB (2017). Matrix bound vesicles and miRNA cargoes are bioactive factors within extracellular matrix bioscaffolds. Neural Regen Res.

[77] van der Merwe Y, Steketee MB (2016). Immunomodulatory approaches to CNS injury: extracellular matrix and exosomes from extracellular matrix conditioned macrophages. Neural Regen Res.

[78] Huleihel, L. *et al*. Matrix-Bound Nanovesicles Recapitulate Extracellular Matrix Effects on Macrophage Phenotype. *Tissue Eng Part A*, 10.1089/ten.TEA.2017.0102 (2017). 10.1089/ten.tea.2017.0102 PMC5689118 28580875

[79] Erzurumlu RS, Jhaveri S, Moya KL, Benowitz LI (1989). Peripheral nerve regeneration induces elevated expression of GAP-43 in the brainstem trigeminal complex of adult hamsters. Brain Res.

[80] Spencer SA, Schuh SM, Liu WS, Willard MB (1992). GAP-43, a protein associated with axon growth, is phosphorylated at three sites in cultured neurons and rat brain. J Biol Chem.

[81] Chong MS (1994). GAP-43 expression in primary sensory neurons following central axotomy. J Neurosci.

[82] Fawcett JW, Keynes RJ (1990). Peripheral nerve regeneration. Annu Rev Neurosci.

[83] Dziki JL, Sicari BM, Wolf MT, Cramer MC, Badylak SF (2016). Immunomodulation and Mobilization of Progenitor Cells by Extracellular Matrix Bioscaffolds for Volumetric Muscle Loss Treatment. Tissue Eng Part A.

[84] Irwin N (2002). Nerve growth factor controls GAP-43 mRNA stability via the phosphoprotein ARPP-19. Proc Natl Acad Sci USA.

[85] Perrone-Bizzozero NI (1991). Post-transcriptional regulation of GAP-43 rnRNA levels during neuronal differentiation and nerve regeneration. Mol Cell Neurosci.

[86] Feldman DE, Brecht M (2005). Map plasticity in somatosensory cortex. Science.

[87] Wu CS, Ballester Rosado CJ, Lu HC (2011). What can we get from ‘barrels’: the rodent barrel cortex as a model for studying the establishment of neural circuits. Eur J Neurosci.

[88] Ebara S, Kumamoto K, Matsuura T, Mazurkiewicz JE, Rice FL (2002). Similarities and differences in the innervation of mystacial vibrissal follicle-sinus complexes in the rat and cat: a confocal microscopic study. J Comp Neurol.

[89] Simons DJ (1978). Response properties of vibrissa units in rat SI somatosensory neocortex. J Neurophysiol.

[90] Simons DJ (1983). Multi-whisker stimulation and its effects on vibrissa units in rat SmI barrel cortex. Brain Res.

[91] Melendez-Vasquez CV (2001). Nodes of Ranvier form in association with ezrin-radixin-moesin (ERM)-positive Schwann cell processes. Proc Natl Acad Sci USA.

[92] Radtke C, Kocsis JD (2012). Peripheral nerve injuries and transplantation of olfactory ensheathing cells for axonal regeneration and remyelination: fact or fiction?. Int J Mol Sci.

[93] Gottschaldt KM, Vahle-Hinz C (1981). Merkel cell receptors: structure and transducer function. Science.

[94] Renehan WE, Munger BL (1986). Degeneration and regeneration of peripheral nerve in the rat trigeminal system. I. Identification and characterization of the multiple afferent innervation of the mystacial vibrissae. J Comp Neurol.

[95] Leiser SC, Moxon KA (2007). Responses of trigeminal ganglion neurons during natural whisking behaviors in the awake rat. Neuron.

[96] Waite PM, Cragg BG (1982). The peripheral and central changes resulting from cutting or crushing the afferent nerve supply to the whiskers. Proc R Soc Lond B Biol Sci.

[97] Shoykhet M, Doherty D, Simons DJ (2000). Coding of deflection velocity and amplitude by whisker primary afferent neurons: implications for higher level processing. Somatosens Mot Res.

[98] Stuttgen MC, Ruter J, Schwarz C (2006). Two psychophysical channels of whisker deflection in rats align with two neuronal classes of primary afferents. J Neurosci.

[99] Rice FL, Mance A, Munger BL (1986). A comparative light microscopic analysis of the sensory innervation of the mystacial pad. I. Innervation of vibrissal follicle-sinus complexes. J Comp Neurol.

[100] Bei F (2016). Restoration of Visual Function by Enhancing Conduction in Regenerated Axons. Cell.

[101] Hong Y (2008). Generating elastic, biodegradable polyurethane/poly(lactide-co-glycolide) fibrous sheets with controlled antibiotic release via two-stream electrospinning. Biomacromolecules.

[102] Marcal H, Ahmed T, Badylak SF, Tottey S, Foster LJ (2012). A comprehensive protein expression profile of extracellular matrix biomaterial derived from porcine urinary bladder. Regen Med.

[103] Freytes DO, Badylak SF, Webster TJ, Geddes LA, Rundell AE (2004). Biaxial strength of multilaminated extracellular matrix scaffolds. Biomaterials.

[104] Brown BN (2010). Surface characterization of extracellular matrix scaffolds. Biomaterials.

[105] Alshadwi, A. & Nadershah, M. Surgical Repair of Trigeminal Nerve Injuries. *A Textbook of Advanced Oral and Maxillofacial Surgery* **3**, 10.5772/64059 (2016).

[106] Charan J, Kantharia ND (2013). How to calculate sample size in animal studies?. J Pharmacol Pharmacother.

[107] McCloy RA (2014). Partial inhibition of Cdk1 in G 2 phase overrides the SAC and decouples mitotic events. Cell Cycle.

