# Supplementary material for: Fetal extracellular matrix nerve wraps locally improve peripheral nerve remodeling after complete transection and direct repair in rat
Source: Sci Rep. 2018 Mar 14;8:4474. doi: 10.1038/s41598-018-22628-8 (PMC5852088; doi:10.1038/s41598-018-22628-8)
Supplement: Supplementary file 1 — DNA gel electrophoresis [file 41598_2018_22628_MOESM1_ESM.docx]

Fetal extracellular matrix nerve wraps locally improve peripheral nerve remodeling after complete transection and direct repair in rat

Tanchen Ren1,2, Anne Faust1,2, Yolandi van der Merwe1,2,4, Bo Xiao6,8, Scott Johnson2,5, Apoorva Kandakatla1,2, Vijay S. Gorantla2,6, Stephen F. Badylak2,5, Kia M. Washington6,7, Michael B. Steketee* 1,2,3

1. Department of Ophthalmology, School of Medicine, University of Pittsburgh, Pittsburgh, PA,
2. McGowan Institute for Regenerative Medicine, University of Pittsburgh, Pittsburgh, PA,
3. Center for Neuroscience, University of Pittsburgh, Pittsburgh, PA,
4. Swanson School of Engineering, Department of Bioengineering, Pittsburgh, PA,
5. Department of Surgery, School of Medicine, University of Pittsburgh, Pittsburgh, PA,
6. Department of Plastic Surgery, School of Medicine, University of Pittsburgh, Pittsburgh, PA,
7. VA Pittsburgh Healthcare System Pittsburgh, PA, USA

8. Plastic Surgery, Xijing Hospital, The Fourth Military Medical Univ., Xi'an, China

*Address correspondence to:

Michael B. Steketee

Department of Ophthalmology and Center for Neuroscience

McGowan Institute for Regenerative Medicine University of Pittsburgh

450 Technology Drive Suite 300

Pittsburgh, PA 15213 USA

Stek0323@gmail.com

Phone: 305-812-4129


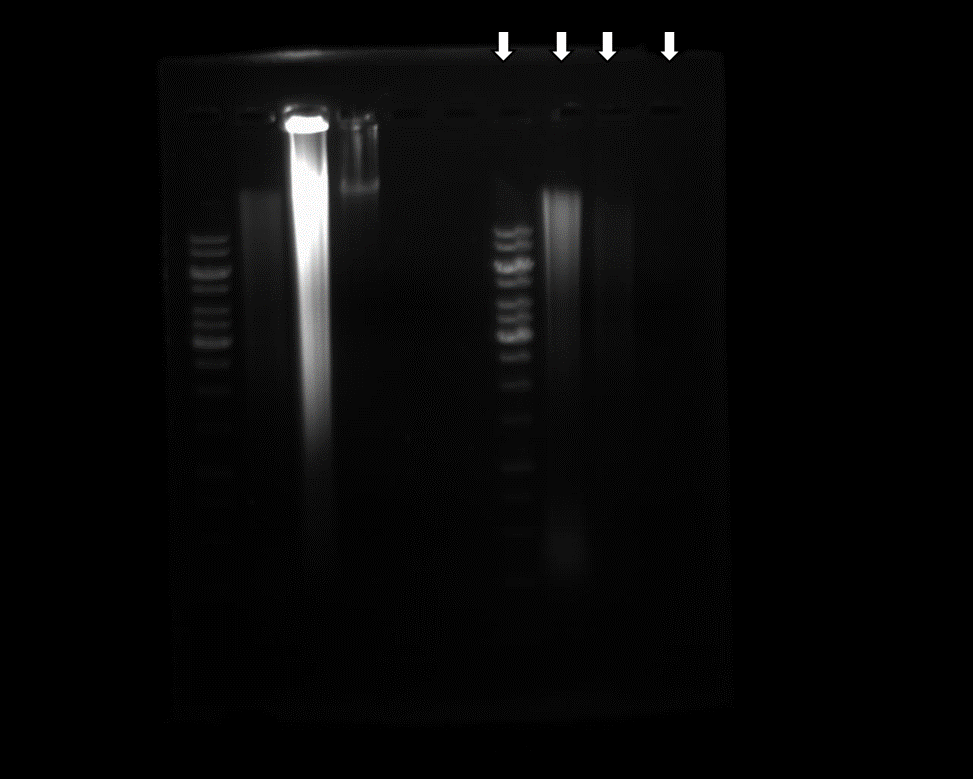


Figure S1 The image of whole gel after DNA electrophoresis experiments. The right 4 lanes (arrowed) indicate the four samples shown in Figure 1c. The 4 lanes at the left represent another tissue decellularization which is not relevant to this article.
